# MicroRNA *miR-378* Regulates Nephronectin Expression Modulating Osteoblast Differentiation by Targeting GalNT-7

**DOI:** 10.1371/journal.pone.0007535

**Published:** 2009-10-21

**Authors:** Shireen Kahai, Shao-Chen Lee, Daniel Y. Lee, Jennifer Yang, Minhui Li, Chia-Hui Wang, Zide Jiang, Yaou Zhang, Chun Peng, Burton B. Yang

**Affiliations:** 1 Sunnybrook Research Institute, Sunnybrook Health Sciences Centre, Toronto, Canada; 2 Department of Biology, York University, Toronto, Canada; 3 Department of Plant Pathology, South China Agricultural University, Guangzhou, China; 4 Division of Life Science, Graduate School at Shenzhen, Tsinghua University, Shenzhen, China; 5 Department of Laboratory Medicine and Pathobiology, University of Toronto, Toronto, Canada; Universität Heidelberg, Germany

## Abstract

MicroRNAs (miRNAs) are small fragments of single-stranded RNA containing 18-24 nucleotides, and are generated from endogenous transcripts. MicroRNAs function in post-transcriptional gene silencing by targeting the 3′-untranslated region (UTR) of mRNAs, resulting in translational repression. We have developed a system to study the role of miRNAs in cell differentiation. We have found that one of the miRNAs tested in our system (miR-378, also called miR-378*) plays a role in modulating nephronectin-mediated differentiation in the osteoblastic cell line, MC3T3-E1. Nephronectin is an extracellular matrix protein, and we have demonstrated that its over-expression enhanced osteoblast differentiation and bone nodule formation. Furthermore, we found that the nephronectin 3′-untranslated region (3′UTR) contains a binding site for *miR-378*. Stable transfection of MC3T3-E1 cells with miR-378 inhibited cell differentiation. MC3T3-E1 cells stably transfected with nephronectin exhibited higher rates of differentiation and nodule formation as compared with cells transfected with nephronectin containing the 3′UTR in the early stages of development, suggesting that endogenous *miR-378* is present and active. However, in the later stages of MC3T3-E1 development, the differentiation rates were opposite, with higher rates of differentiation and nodule formation in the cells over-expressing the 3′UTR of nephronectin. This appeared to be the consequence of competition between nephronectin and UDP-N-acetyl-alpha-D-galactosamine:polypeptide N-acetylgalactosaminyltransferase 7 (GalNAc-T7 or GalNT7) for *miR-378* binding, resulting in increased GalNT7 activity, which in turn lead to increased nephronectin glycosylation and product secretion, thereby resulting in a higher rate of osteoblast differentiation.

## Introduction

Over the past few years, microRNAs (miRNAs) have emerged as a prominent class of gene regulatory factors [1]. MiRNAs are single-stranded RNAs of 18–24 nucleotides in length, and are generated from endogenous transcripts producing hairpin structures by an RNase III-type enzyme [2]–[5]. miRNA functions as a regulator in gene silencing by partially complementing with the 3′-untranslated region (3′UTR) of target mRNAs, leading to translational repression [6]–[8]. By silencing various target mRNAs, miRNAs have key roles in various regulatory pathways. This involves cell proliferation [9], [10], division [11], [12], apoptosis [13], [14], cell differentiation [15]–[20], tissue development [21]–[27], tumor formation [28]–[43], protein expression [44]–[46], immuno-response [47], and viral infection [48]–[51]. Although miRNAs have emerged as key regulators of gene expression, our understanding of the specific roles of miRNAs has been limited due to the difficulty in tracking the functions of a particular miRNA. Furthermore, since chemically synthetic miRNAs are easily degraded, it is impossible to obtain stable cell lines expressing miRNAs for long-term functional analysis in vitro and in vivo. Although expression of a large DNA fragment has made stable expression possible [52], in many cases miRNAs are expressed as a cluster, making it difficult to distinguish the function of a particular miRNA from others. To allow long-term studies of miRNA functions in vitro and in vivo, we have developed an expression vector expressing two copies of pre-miRNAs, a green fluorescent protein (GFP) tracking unit, and an antibiotic selection marker [53], [54]. This allows stable expression of double amounts of the miRNA of interest in cells for functional studies.

Nephronectin was discovered in the developing mouse kidney as a novel ligand for the integrin α8β1. It is a 70–90 kDa secreted extracellular matrix protein that contains a putative signal peptide at the N-terminus, five epidermal growth factor (EGF)-like repeats (amino acids 57–250), an RGD sequence (amino acids 382–384), and a C-terminal MAM domain (amino acids 417–561). As nephronectin is expressed in a variety of tissues in the developing mouse embryo, we studied the role of nephronectin in bone development. We also examined the regulation of nephronectin expression by *miR-378* and demonstrated that *miR-378* up-regulated nephronectin expression, and enhanced nephronectin glycosylation and secretion via binding to the 3′UTR of nephronectin mRNA. Interaction of *miR-378* with nephronectin 3′UTR arrested this miRNA and freed another *miR-378* target GalNT7, an enzyme essential for nephronectin glycosylation. As a consequence, nephronectin glycosylation was enhanced and osteoblast differentiation was promoted.

## Results

### Nephronectin promotes osteoblast differentiation

**Figure 1:**
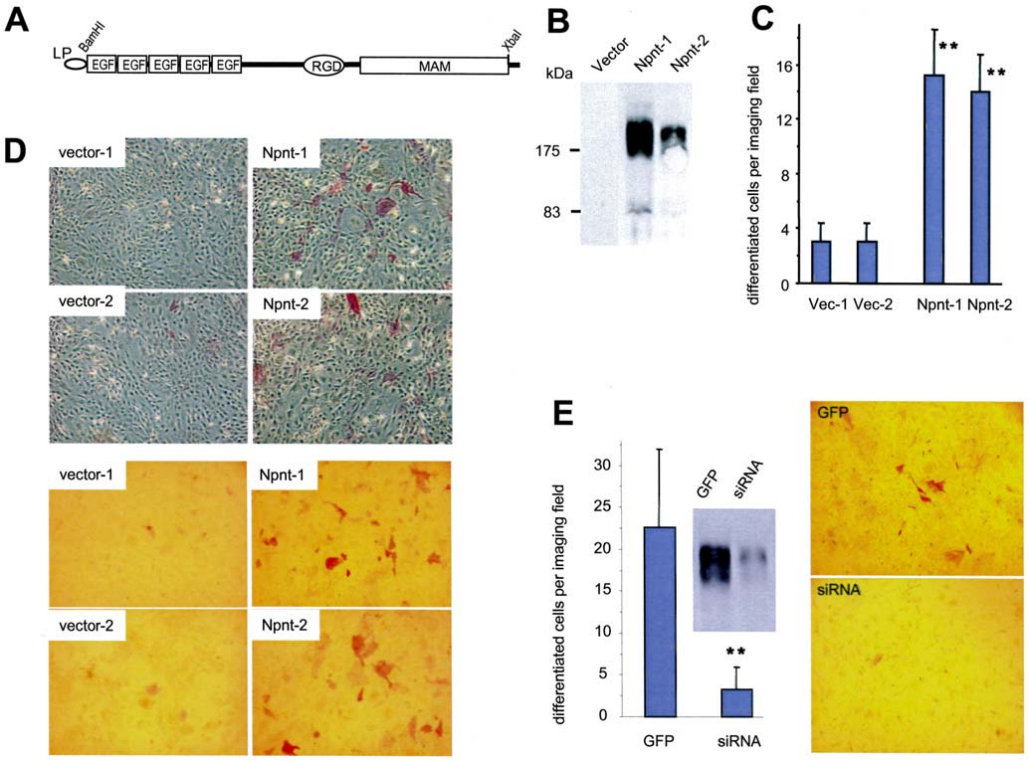
Alkaline phosphatase expression in MC3T3-E1 cells over-expressing nephronectin. (A) A full-length nephronectin construct was generated containing the 5 EGF-like repeats, RGD domain, and the MAM domain. A leading peptide (LP) was tagged at the N-terminal region of the construct (Panel A). (B) After transfection, conditioned medium from two nephronectin- and one vector-transfected MC3T3-E1 clones was analyzed for stable expression and secretion of the full-length nephronectin protein by western blot, using the monoclonal antibody 4B6 which recognizes an epitope in the leading peptide. (C) Two nephronectin- and two vector-transfected MC3T3-E1 clones were grown for 10 days and stained for ALP expression. Multiple fields were examined for each construct, and stained cells were counted per field for a quantitative graphical representation. Over-expressing nephronectin (Npnt-1 and Npnt-2) enhanced ALP expression. (D) Typical micrographs of ALP expression in the two clones were taken using two different phases (top and bottom panels) of the microscope for better estimation of ALP activity. (E) Left, MC3T3-E1 cells stably transfected with an siRNA construct against nephronectin were grown in parallel with cells transfected with the empty vector for 10 days and then stained for ALP activity. Multiple fields were examined for each construct, and stained cells were counted per field for quantitative analysis. Inset shows the expression level of the nephronectin protein after transfection with the siRNA against nephronectin. Right, typical micrographs of ALP expression are shown.

To study the role of nephronectin during osteoblast development, we generated a nephronectin construct (Npnt). A leading peptide (LP) of chicken link protein was tagged at the N-terminal region, which contains the secretion signal and an epitope recognized by the monoclonal antibody 4B6 (Fig. 1A). The osteoblast cell line MC3T3-E1 was stably transfected with the nephronectin construct or an empty vector. Conditioned medium from each stable cell line was examined for expression and secretion of nephronectin by western blot using the 4B6 antibody (Fig. 1B). We analyzed the activity of alkaline phosphatase (ALP), a well known marker of commitment to the osteoblast lineage, in the nephronectin-transfected MC3T3-E1 cells and observed a significantly higher level of ALP expression in the cells over-expressing nephronectin as compared with the vector-transfected cells (Fig. 1C). Typical micrographs are provided to show cell morphology after staining (ALP activity seen as the red colour in the wells, Fig. 1D, upper panel). Using a different phase on the microscope, we could focus on only the cells exhibiting ALP activity due to their red staining (Fig. 1D, lower panel). This allowed quantification of the stained cells. Our results indicated that over-expression of nephronectin promotes osteoblast differentiation.

To determine the effect of endogenous nephronectin on MC3T3-E1 cell differentiation, we analyzed the effect of knocking down nephronectin expression on osteoblast differentiation. Cell lines stably transfected with a small interfering RNA (siRNA) plasmid targeting nephronectin (Npnt-siRNA), or with the GFP vector alone, were generated. The GFP- and Npnt-siRNA-expressing cells were cultured under nodule-inducing conditions and then stained for ALP expression to identify differentiating cells. We observed minimal ALP expression in the MC3T3-E1 cells over-expressing the siRNA as compared with the vector control (Fig. 1E). Our results indicated that down-regulation of endogenous nephronectin expression inhibited osteoblast differentiation.

### The effect of nephronectin 3′UTR on osteoblast differentiation

**Figure 2:**
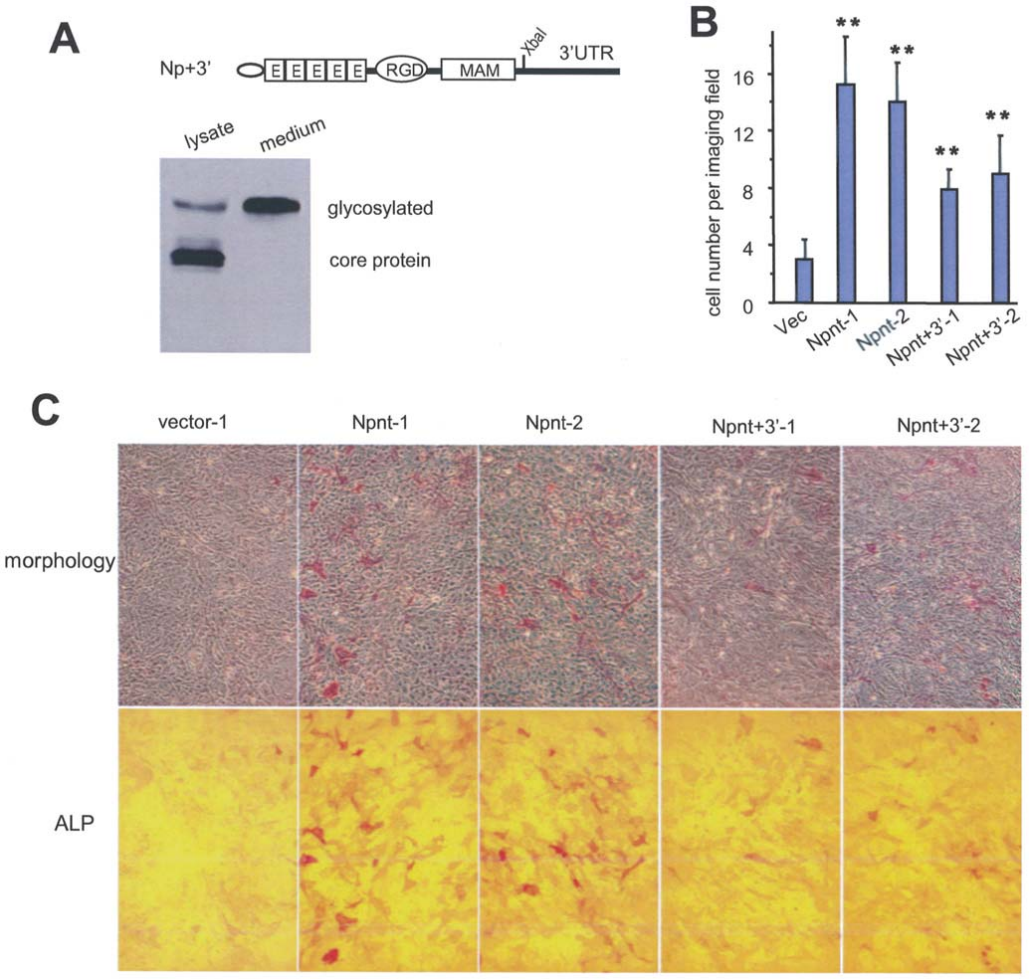
The effect of nephronectin 3′UTR on osteoblast differentiation. (A) A nephronectin construct with a fragment of the nephronectin 3′UTR spanning the target sequence of miR-378 is shown (Npnt+3′). Cell lysate and conditioned medium from MC3T3-E1 cells stably transfected with Npnt+3′ were analyzed by western blot showing secretion of the product. (B) Cells transfected with the full-length nephronectin (Npnt-1 and Npnt-2), nephronectin containing the 3′UTR (Npnt+3′-1 and Npnt+3′-2), or with the control vector were grown for 10 days and stained for ALP expression. Multiple fields were examined for each construct, and stained cells were counted per field for quantitative analysis. (C) Typical micrographs of ALP expression are shown using two different phases (top and bottom panels) of the microscope.

We then studied regulation of nephronectin expression by miRNAs, as miRNAs have been reported to play essential roles in cell differentiation. We generated a nephronectin expression construct with a fragment of the nephronectin 3′UTR (Fig. 2A, Npnt+3′). MC3T3-E1 cells were transfected with the construct Npnt+3′ to generate stable cell lines. Conditioned medium was examined for expression and secretion of the ectopic nephronectin by western blot (Fig. 2A). MC3T3-E1 cells transfected with either the Npnt construct, the Npnt+3′ construct, or the GFP vector control were cultured for 6 days under nodule-inducing conditions, followed by staining for ALP activity to identify differentiating osteoblasts. Again, over-expression of nephronectin promoted osteoblast differentiation as seen by the significantly higher ALP staining as compared with the control cells (Fig. 2B and C). A much lower rate of osteoblast differentiation was observed in the cells expressing the Npnt+3′ construct (Npnt+3′-1 and Npnt+3′-2), thereby providing further evidence towards the post-transcriptional repression of nephronectin by endogenous miRNAs during osteoblast differentiation.

**Figure 3:**
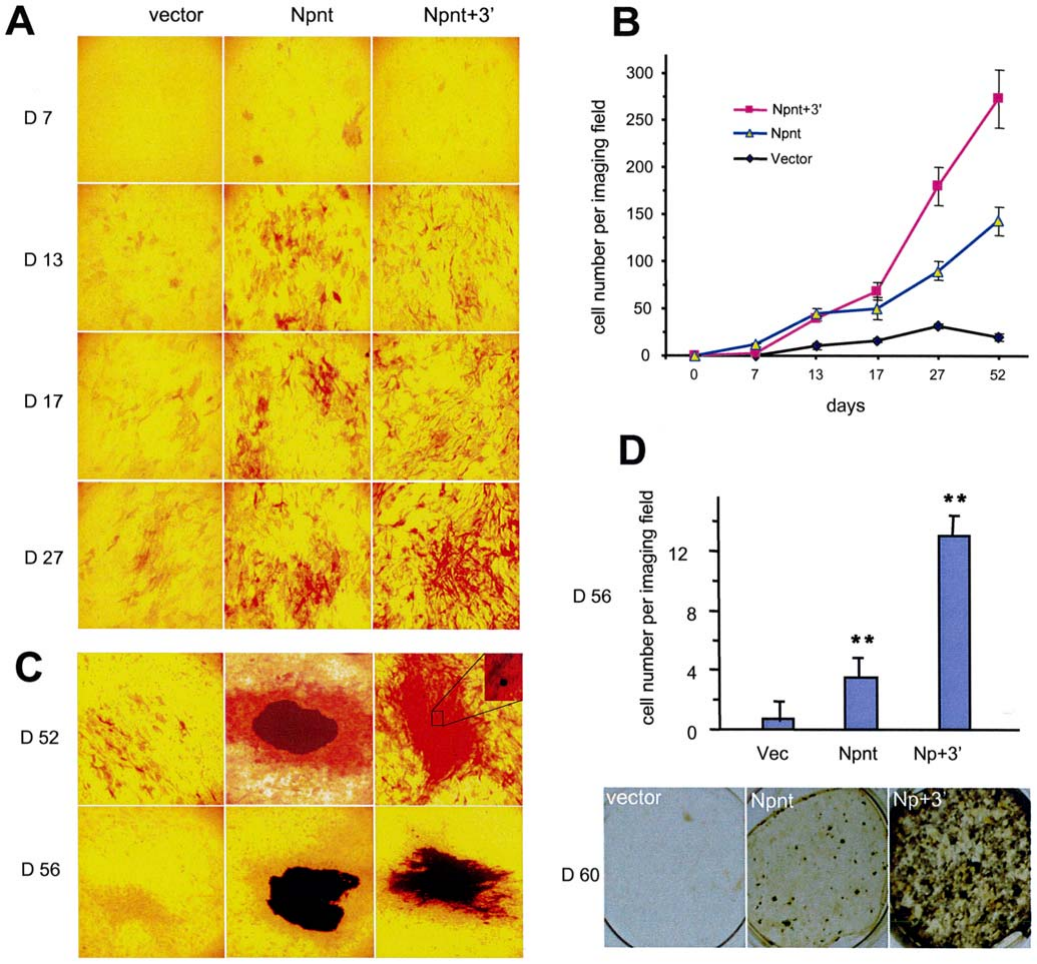
Nephronectin 3′UTR promotes nodule formation. (A) Time course analysis showing the different effects of Npnt and Npnt+3′ transfection on MC3T3-E1 differentiation: early stages, Npnt>Npnt+3′; late stages, Npnt<Npnt+3′. (B) Multiple fields were examined for each construct, and stained cells were counted per field for quantitative analysis. (C) The cell lines were cultured under nodule-inducing conditions over the three different phases of osteoblast development. Magnified images of bone nodules formed on days 52 and 56 are shown after silver nitrate staining for the detection of bone nodules. (D) Photographs of the whole plates showing bone nodules on day 60 are shown in the lower panel. The total number of bone nodules was statistically analyzed and have been represented in the upper panel. ** p<0.01.

We then investigated the effect of over-expressing the 3′UTR of nephronectin over the full course of osteoblast development, including mineralization and nodule formation. The transfected cells were grown in parallel in nodule inducing conditions (regular growth medium supplemented with ascorbic acid, beta-glycerophosphate, and dexamethasone) over the three phases of bone development (proliferation, differentiation, and mineralization/nodule formation). ALP expression and nodule formation were analyzed on different days over the full course of osteogenesis. Consistent with the above results, the Npnt-transfected cells showed a significantly higher rate of differentiation as compared with the vector controls (Fig. 3A). The Npnt+3′-transfected cells exhibited a lower rate of differentiation than the Npnt-transfected cells during the early stages of osteogenesis until day 13. The rates of differentiation became similar between the Npnt- and Npnt+3′-transfected cells by day 17, and finally, during the later stages of osteogenesis (after day 27) the Npnt+3′-transfected cells displayed much higher levels of ALP activity than the Npnt-transfected cells. Photographs of the whole wells are provided in Supplementary (Fig. S1A). Statistical analysis indicated that all differences were significant (Fig. 3B).

Once we observed nodule formation, the cells were stained with silver nitrate for visualization of bone nodules. Multiple nodules were first seen in the nephronectin-transfected cells on day 52 after plating, indicating that the faster and greater rates of differentiation in these cells led to faster and more pronounced nodule formation as compared with the controls. The Npnt+3′-transfected cells exhibited mineralization with minimal nodule formation on day 52 (Fig. 3C). Interestingly, although the Npnt+3′-transfected cells exhibited delayed nodule formation compared with the nephronectin-transfected cells, by day 56, the Npnt+3′-transfected cells exhibited fully formed nodules in a significantly higher density than the nephronectin-transfected cells (Fig. 3D, upper panel, Fig. S1B). By day 60, the Npnt+3′ plates were almost fully filled with bone nodules (Fig. 3D, lower panel).

### The effect of 3′UTR on nephronectin glycosylation

**Figure 4:**
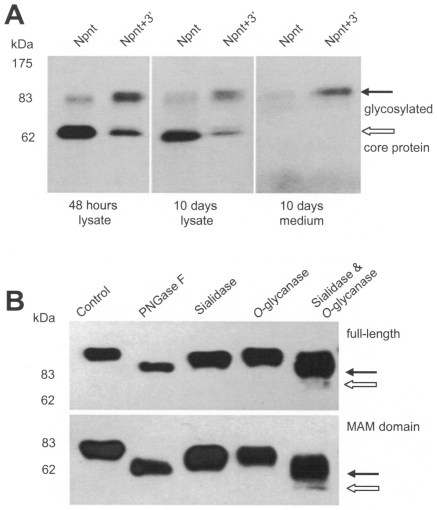
The 3′UTR of nephronectin promoted nephronectin protein glycosylation. (A) Cell lysate from the two MC3T3-E1 stable cells lines (over-expressing the nephronectin protein with and without the 3′UTR) was prepared from 48 hour cultures and 10 day cultures. The lysate and conditioned medium was analyzed on western blot probed with the 4B6 antibody. The bands representing glycosylated nephronectin and the nephronectin core protein have been marked separately. (B) Conditioned medium was treated with different de-glycosylation enzymes to reduce the observed molecular size in western blot to confirm nephronectin glycosylation. The cells transfected with either the full-length nephronectin or just the MAM domain possessed potential *O*-glycosylation, which accounts for the significant size-difference in Fig. 4A. Filled arrows indicate the de-*O*-glycosylated product; and open arrows show the size of core protein.

We then investigated whether or not the increase in differentiation and nodule formation seen in the Npnt+3′-transfected cells was associated with nephronectin production. Npnt- and Npnt+3′-transfected cell lines were cultured in parallel under nodule inducing conditions. One set of cells was lysed and the protein was extracted after 48 hours (just before confluence), while the other set was lysed for protein extraction after 10 days. Both sets were analyzed for nephronectin levels by western blot. Forty-eight hours after induction, the level of glycosylated nephronectin was found to be much lower than the level of core protein in the Npnt-transfected cells, while the Npnt+3′-transfected cells containing the 3′UTR showed much greater levels of glycosylated protein than core protein (Fig. 4A). Ten days after induction, the level of secreted glycosylated protein was significantly greater in the Npnt+3′-transfected cells.

It has been predicted that a high degree of glycosylation occurs in the region of nephronectin containing the MAM domain [55]. Therefore, to confirm glycosylation of nephronectin, a truncated nephronectin construct was generated containing only the MAM domain. MC3T3-E1 cells were transfected separately with either the construct containing only the MAM domain or the full-length nephronectin. The conditioned medium obtained from the MAM and the Npnt-transfected cells was dialyzed and subjected to treatments with PNGase F, sialidase, *O*-glycanase, and a mix of sialidase and *O*-glycanase. A reduction in molecular size was obtained with PNGase F treatment, and treatment with a mix of sialidase and *O*-glycanase (Fig. 4B). De-glycosylation of nephronectin by various enzymes indicated its potential *N*- and *O*-glycosylation. The major band shift by co-treatment with sialidase and *O*-glycanase suggested its glycoform as sialic acid-capped *O*-glycosylation.

### Effect of *miR-378* on osteoblast differentiation

**Figure 5:**
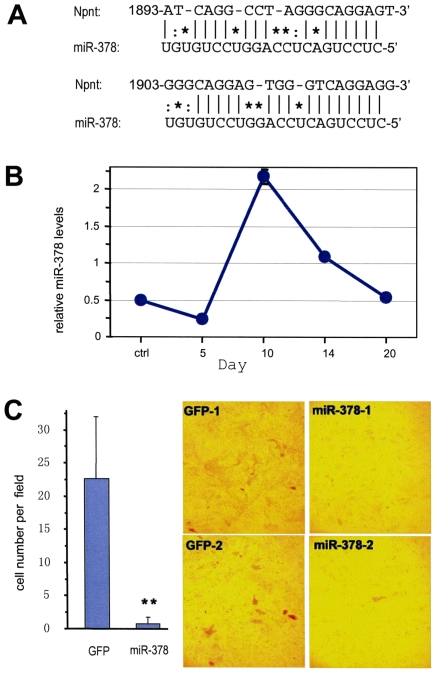
Targeting of nephronectin with miR-378. (A) Computational algorithms were used to predict the miRNA that binds to a target sequence in the 3′-UTR of nephronectin. miR-378 was found to have the strongest binding to the 3′UTR of nephronectin from nucleotides 1902–1923. (B) Expression of endogenous miR-378 in MC3T3-E1 cells under differentiation-inducing conditions was measured by real-time PCR. (C) Left, MC3T3-E1 cells stably transfected with the miR-378 construct were grown in parallel with cells transfected with the control vector for 10 days and stained for ALP activity. Multiple fields were examined for each construct, and stained cells were counted per field for quantitative analysis. Right, typical micrographs of ALP expression are shown in two separate stable clones.

The above results indicate that the 3′UTR of nephronectin played a role in modulating nephronectin-mediated osteoblast differentiation. We then searched for potential miRNAs that might regulate nephronectin functioning. Multiple computational approaches were used to analyze the 3′UTR of nephronectin mRNA for miRNA binding sites/sequences. The miRNA that was predicted to be of great potential for nephronectin binding was *miR-378*. *MiR-378* base pairs well with the 3′UTR of nephronectin from nucleotides 1893–1913 and 1903–1923 (Fig. 5A), and was therefore selected for further analyses as a putative regulator of nephronectin during osteoblast development. Expression of endogenous *miR-378* was examined in MC3T3-E1 osteoblasts under differentiation-inducing conditions. The experiments indicated that *miR-378* expression increased significantly during cell condensation at around day 10, followed by a sharp decline during cell differentiation (Fig. 5B), suggesting involvement of *miR-378* in MC3T3-E1 cell differentiation.

To study the role of *miR-378* in MC3T3-E1 differentiation, it is essential to ectopically provide the cells with *miR-378* constantly. Current methods use chemically synthetic miRNAs for functional studies, which could only last for a few days before degradation. Since nephronectin-mediated MC3T3-E1 cell differentiation takes place at least one week after induction, it is essential to obtain stable cell lines expressing *miR-378*. We thus developed a method to generate a construct that stably expresses miR-378. The construct contained a H1 promoter driving expression of pre-miR-378, an antibiotic selection marker, and a GFP for monitoring transfection efficiency [53]. Transient transfection of U87 cells with this construct indicated high efficiency of GFP expression. Using two primers, miR-378N (5′agatctagggctcctgactcc) and miR-378C (5′aggccttctgactccaagtcc), which amplify the exogenously transfected miR-378 but not endogenous *miR-378*, we observed expression of pre-miR-378 (data not shown). This pre-miR-378 was processed to produce mature miR-378 as expected (data not shown). This expression system has been successfully used in our previous studies [53], [54].

MC3T3-E1 cells were stably transfected with the miR-378 construct or a control vector expressing GFP alone. Two sets of clones were cultured in parallel under nodule-inducing conditions. The cells were cultured for 6 days and then stained for the expression of ALP to identify differentiating cells. We observed minimal ALP expression in the MC3T3-E1 cells over-expressing miR-378 as compared with the two empty vector-transfected cell lines. Typical photographs are shown (Fig. 5C, right). Using a different phase on the same microscope, only the cells stained for ALP were visualized and counted for quantification of ALP expression (Fig. 5C, left). Based on the significant difference in ALP expression between MC3T3-E1 cells transfected with the empty vector and those expressing miR-378, our results indicate that over-expression of miR-378 inhibits osteoblast differentiation.

### Up regulation of nephronectin expression by *miR-378*

**Figure 6:**
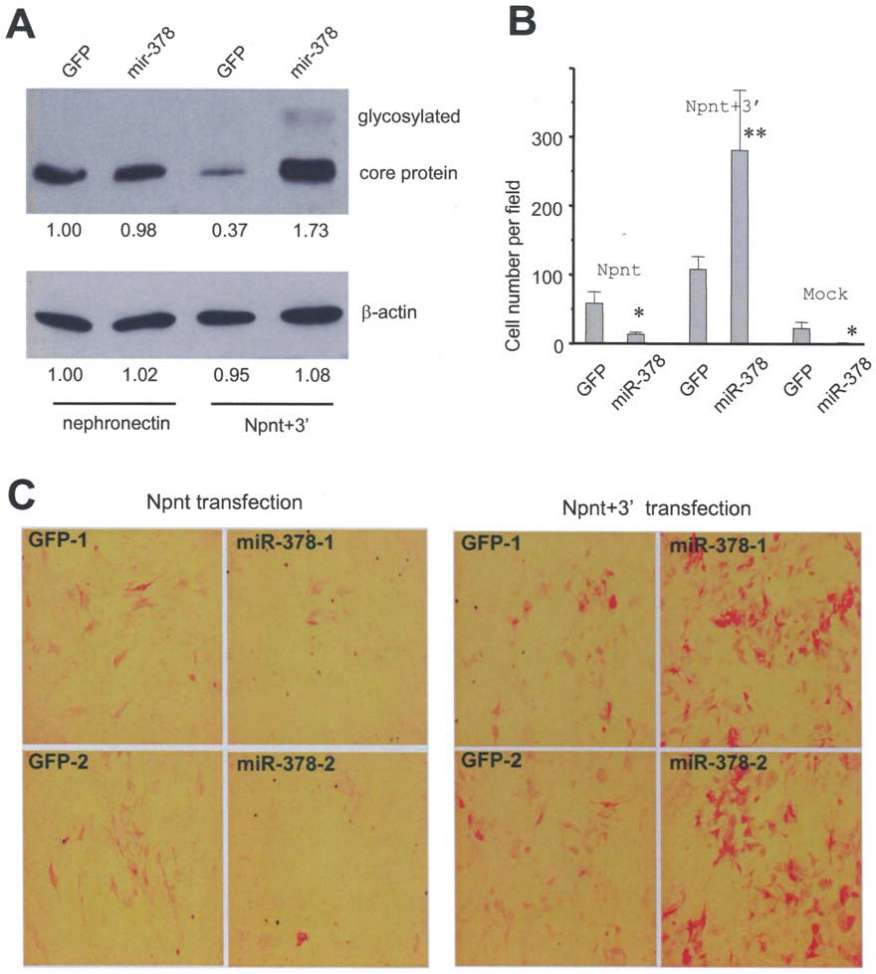
Effect of miR-378 and 3′UTR on nephronectin expression, and its biological activity in double-transfected cells. (A) Western blot for Npnt expression in the double-transfected cells. miR-378 elevated Npnt expression only when 3′UTR was present. Both glycosylated and core protein bands were observed. (B) Cell differentiation as revealed by ALP staining for Npnt-transfected and Npnt+3′-transfected cells. miR-378 expression inhibited differentiation in Npnt- and mock-transfected cells. However, miR-378 promoted osteoblast differentiation extensively in Npnt+3′-transfected cells, implying that miR-378 interacted with the 3′UTR and exerted its effect. (C) Typical results in Panel B are provided showing that the presence of the 3′UTR promoted cell differentiation upon miR-378 transfection.

It has been known that miRNAs repress mRNA translation by targeting the 3′UTR. We performed experiments to test whether co-transfection of Npnt+3′ with miR-378 would reduce ectopic nephronectin expression. Unexpectedly, we not only detected an increase in the levels of nephronectin glycosylation, but also observed an increased level of nephronectin core protein in MC3T3-E1 osteoblasts stably transfected with Npnt+3′ and miR-378 (Fig. 6A). This result was then confirmed using multiple transient and stable transfections of HEK293 cells (data not shown).

To better understand the increase in nephronectin production by *miR-378* expression, we performed differentiation experiments in the same combination as those performed in the western blot analyses. Upon miR-378 transfection, differentiation in nephronectin-transfected cells was inhibited in a similar manner as in the mock-transfected cells (Fig. 6B). Co-expression of miR-378 with Npnt+3′ significantly promoted cell differentiation, consistent with enhanced nephronectin synthesis, glycosylation, and product secretion. The Npnt+3′-transfected cells not only induced a greater number of cells to undergo differentiation, but also produced stronger ALP staining seen by the red color (Fig. 6C). To corroborate these results, we carried out proliferation experiments on the MC3T3-E1 cells stably co-transfected with miR-378/GFP and nephronection/Npnt+3′. The experiments showed that co-transfection of miR-378 and nephronectin increased cell proliferation (Fig. S1C), while co-transfection of miR-378 and Npnt+3′ inhibited cell proliferation (Fig. S1D) as compared with the control groups.

**Figure 7:**
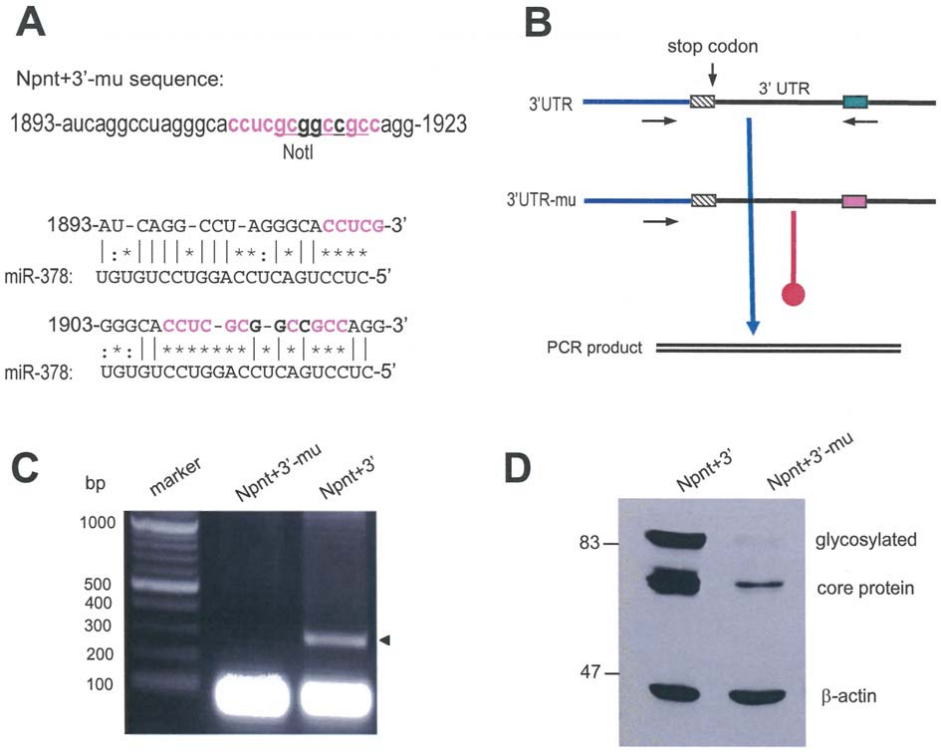
Mutation of the miR-378 binding sites inhibits nephronectin glycosylation. (A) Mutation was generated in the 3′UTR of Npnt+3′ construct as shown (Npnt+3′-mu, upper panel). This impaired both putative binding sites for miR-378 (bottom). (B) Strategy of PCR showing interaction of miR-378 with Npnt 3′UTR, which was abolished by sited-directed mutagenesis. (C) Typical PCR products are shown using the mature miR-378 as a primer for the PCR (arrow). The lower bands were primers used for the PCR assays. (D) Cell lysate prepared from cells transfected with Npnt+3′ and the mutant Npnt+3′-mu was analyzed with western blot showing decreased expression of the mutant construct.

To test the significance of the putative binding sites on nephronectin expression, we generated mutations on both binding sites to disrupt the base pairing structures between miR-378 and nephronectin 3′UTR (Fig. 7A). PCR assays were used to test the potential interactions as previously described [56]. Mature miR-378 was used as a primer in the PCR paring with another primer located in the vector. A plasmid (3′UTR) containing a small fragment of Npnt coding sequence and the 3′UTR and a plasmid (3′UTR-mu) containing the mutation as shown in Fig. Fig. 7A served as PCR-templates (Fig. 7B). PCR product was produced with 3′UTR as a template but could not be detected with 3′UTR-mu as a template (Fig. 7C). Cells were also transfected with nephronectin containing the 3′UTR (Npnt+3′) or the mutant 3′UTR (Npnt+3′-mu). Expression of the mutant construct Npnt+3′-mu produced lower levels of core protein and secreted product (Fig. 7D), suggesting that the miR-378 binding sites are important for the upregulation of nephronectin production and secretion.

### The 3′UTR of nephronectin modulates *miR-378* targeting GalNT7

**Figure 8:**
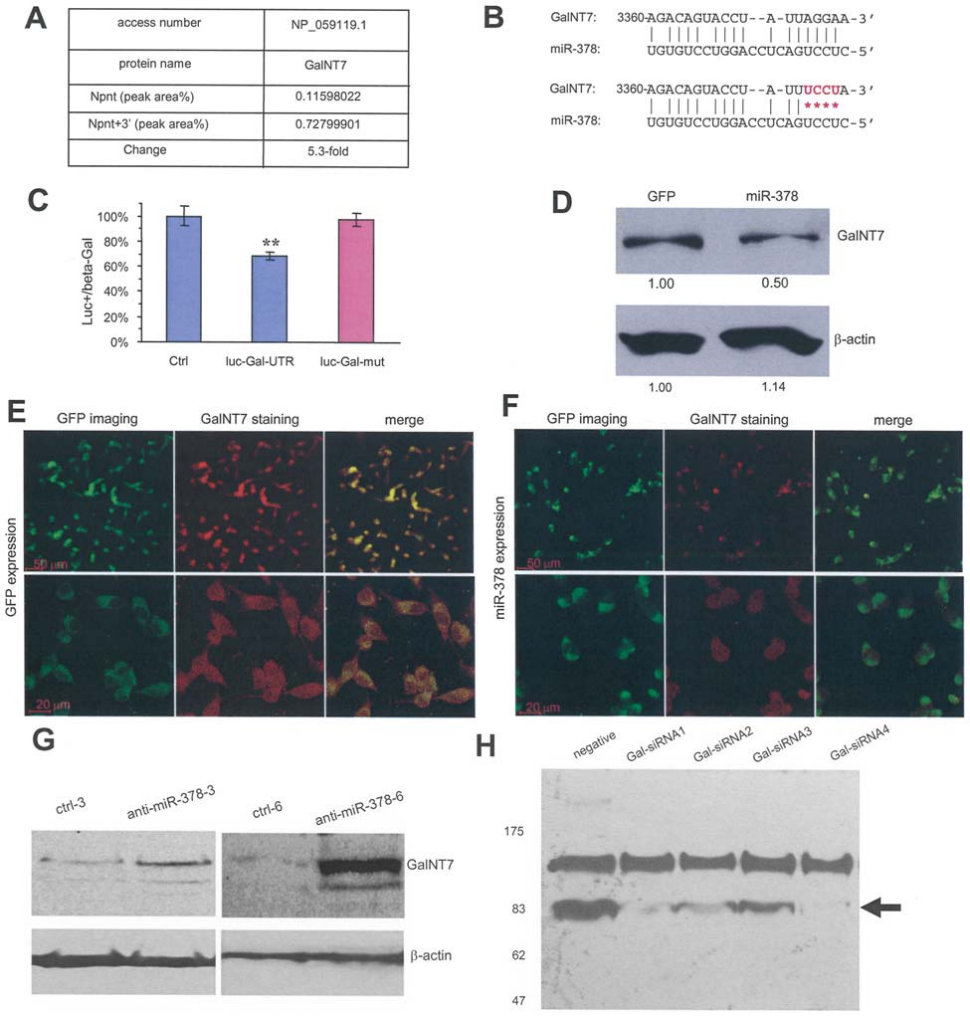
Targeting of *O*-glycosyltransferase, UDP-*N*-acetyl-alpha-*D*-galactosamine:polypeptide *N*-acetylgalactosaminyltransferase 7 (GalNT7) by miR-378. (A) U87 cells transfected with Npnt or Npnt+3′ were harvested and subjected to proteomic analysis. Up-regulation of GalNT7 expression was seen in cells transfected with Npnt+3′. (B) Computational algorithms were used to predict potential targeting of miR-378 on GalNT7. miR-378 was found to possesses a potential target site on GalNT7 in nucleotides 3360-3378. A fragment of the GalNT7 3′UTR harbouring the miR-378 target site was inserted into a luciferase reporter vector. The seed region for miR-378 binding was mutated in GalNT7 as shown (red). (C) Luciferase activity assays were performed by co-transfection of miR-378 with luciferase reporter constructs. Decreased luciferase activity was detected in the construct containing GalNT7 3′UTR, which was abolished when the miR-378 target site was mutated. (n = 4, ** p<0.01). (D) Western blot showing decreased expression of GalNT7 in miR-378-transfected cells compared with GFP-transfected cells. (E-F) U87 cells stably transfected with GFP (E) or miR-378 (F) were immunostained with anti-GalNT7 antibody, followed by confocal microscopic visualization. While both types of cells expressed GFP (green), miR-378-transfected cells expressed lower levels of GalNT7 than the GFP-transfected cells (red). As a result, the merged color of miR-378-transfected cells were less yellow. (G) MC3T3 cells were transfected with anti-miR-378 or a control vector at 3 or 6 µg plasmids. Cell lysates were prepared and analyzed on western blot probed with anti-GalNT7 antibody. Transfection with anti-miR-378 enhanced GalNT7 expression. (H) MC3T3 cells were co-transfected with nephronectin and one of the four siRNA oligos against GalNT7 or an oligo at random sequence serving as a negative control. Culture medium was analyzed on western blot probed with the monoclonal antibody 4B6 that recognizes nephronectin. Transfection with siRNAs against GalNT7 decreased the levels of secreted nephronectin.

The initial delay and later increase in the onset of differentiation and nodule formation seen in the Npnt+3′-transfected cells as compared with the nephronectin-transfected cells suggests that the nephronectin 3′UTR is significant in osteoblast differentiation. We then examined protein expression affected by the 3′UTR. HEK293 cells stably transfected with nephronectin or nephronectin containing the 3′UTR were harvested and subjected to proteomic analysis performed by WEMB Biochem Inc (Richmond Hill, ON). A large number of proteins were altered (Table S1). Interestingly, expression of UDP-N-acetyl-alpha-D-galactosamine:polypeptide N-acetylgalactosaminyltransferase 7 (GalNT7), an enzyme known to glycosylate proteins, in the Npnt+3′-transfected cells increased to 5.3-fold as compared with nephronectin-transfected cells [fold change = (reading ^Npnt+3′^ – reading ^nephronectin^)/reading ^nephronectin^] (Fig. 8A).

To unravel the mechanism, we undertook computational approaches and identified a putative binding site for *miR-378* at GalNT7 (Fig. 8B). The GalNT7 3′UTR containing the miR-378 target site was inserted into the luciferase reporter vector producing a construct named luc-Gal-UTR (Fig. S2A). The miR-378 target site was mutated producing a mutant construct named luc-Gal-mut. Luciferase activity assays were performed to confirm the targeting. U343 cells were co-transfected with miR-378 and luc-Gal-UTR or luc-Gal-mut. Figure 8C shows that miR-378 significantly decreased luciferase activity in luc-Gal-UTR-transfected cells, but did not affect luciferase activity in the luc-Gal-mut-transfected cells, indicating targeting of GalNT7 3′UTR by miR-378.

To confirm the down-regulation of GalNT7 by *miR-378*, cell lysate was prepared from the cells transfected with miR-378 or GFP and analysed by western blot. Repression of GalNT7 was detected in the cells transfected with miR-378 as compared with the cells transfected with GFP (Fig. 8D). U87 cells transfected with GFP (Fig. 8E) or miR-378 (Fig. 8F) were subjected to immunocytometry probed with anti-GalNT7 antibody, followed by confocal microscopic examination. MiR-378-transfected cells expressed lower levels of GalNT7 than the GFP-transfected cells. These results were also confirmed in MT-1 cell line (Fig. S2B).

On the other hand, we transfected MC3T3 cells, which express high level of endogenous miR-378 as demonstrated above, with anti-miR-378 expression construct or a control vector. Cell lysates were prepared and analyzed on western blot probed with anti-GalNT7 antibody. The experiments showed that GalNT7 expression was promoted by anti-miR-378 transfection (Fig. 8G), suggesting that miR-378 is sufficient in regulating GalNT7 expression. Furthermore, we transfected MC3T3 cells with siRNAs against GalNT7 designed and synthesized by GenePharma (Shanghai). We found that all four siRNAs significantly silenced GalNT7 expression (Fig. S2C). The effects of these siRNAs on nephronectin post-translational modification and secretion were tested by co-transfecting the MC3T3 cells with one of these four siRNA or an RNA oligo of random sequence with nephronectin expression construct. We found that co-transfection with the siRNAs against GalNT7 produced lower levels of nephronectin in the culture medium as compared with the transfection using the RNA oligo with random sequence (Fig. 8H, arrow), suggesting decreased secretion of nephronectin product. Since glycosaminoglycan of a core protein by modifying enzymes is essential for proteoglycan secretion [57]–[59], these results confirm that GalNT7 plays a role in nephronectin synthesis. We also detected a band at higher mass (∼100 kDa) that is unaffected by siRNA expression, suggesting that GalNT7 is not involved in the modification of this band.

**Figure 9:**
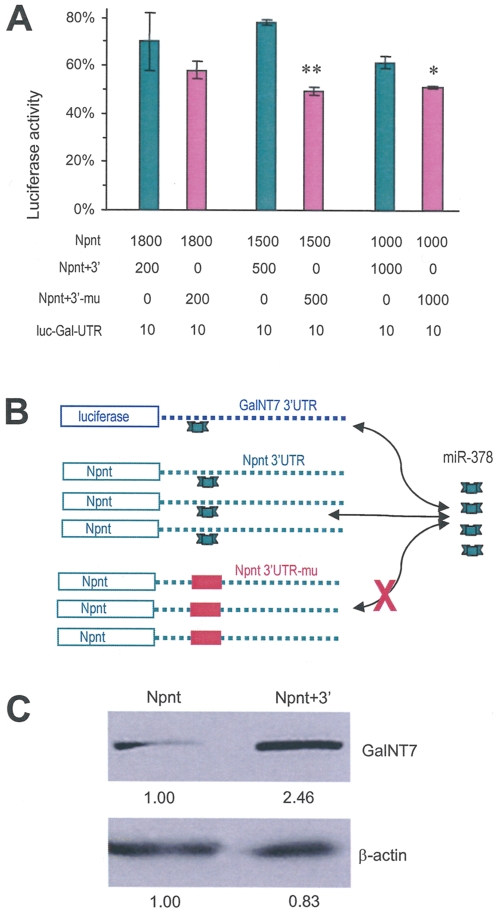
Npnt 3′UTR competes with GalNT7 3′UTR for miR-378 binding. (A) U343 cells were transiently transfected with luciferase reporter vector harboring the GalNT7 3′UTR (lu-Gal-UTR) and different constructs at the amounts indicated on the figure (ng). Luciferase activities were normalized using the control as 100%. The luciferase activities were higher when co-transfected with Npnt+3′ as compared with Npnt+3′-mu, suggesting that increased supplies of Npnt+3′ absorbed some endogenous miRNAs freeing luciferase translation. (B) Diagram showing competition of Npnt+3′, but not Npnt+3′-mu, with GalNT7 for miR-378 binding. As a result, luc-Gal-UTR was freed from miR-378 binding and thereby having higher luciferase activities. (C) Western blot showing increased expression of GalNT7 in the Npnt+3′-transfected cells compared with the Npnt-transfected cells.

We reasoned that over-expressing the 3′UTR-containing nephronectin mRNA resulted in greater binding capacity of nephronectin 3′UTR to miR-378 and lesser concentrations than usual of free miR-378 that would be available for targeting and binding to GalNT7. This would free more GalNT7 mRNA and up-regulate GalNT7 protein levels for enhancing glycosylation of nephronectin. As a consequence, due to more than usual glycosylation of the core protein, secretion of nephronectin product would be promoted. To test this, competition assays were performed. The luciferase reporter vector containing the 3′UTR of GalNT7 was co-transfected with Npnt+3′ or Npnt+3′-mu. Analysis of luciferase activities indicated that luc-Gal-UTR expressed greater activities when co-transfected with Npnt+3′ than when co-transfected with Npnt+3′-mu (Fig. 9A). As shown in the diagram (Fig. 9B), Npnt 3′UTR was able to bind miR-378 and free GalNT7 3′UTR. As a result, luc-Gal-UTR activities increased. On the other hand, Npnt 3′UTR-mu could no longer bind miR-378, nor up-regulate luc-Gal-UTR activities.

To confirm these results, we prepared cell lysate from cells transfected with nephronectin or Npnt+3′ and subjected them to western blot analysis, probing them with anti-GalNT7 antibody (Abnova, Taipei). The results indicated that cells transfected with Npnt+3′ expressed higher levels of GalNT7 than cells transfected with nephronectin (Fig. 9C). The experiments concluded that the presence of nephronectin 3′UTR arrested miR-378 and freed GalNT7 mRNA for GalNT7 protein expression.

## Discussion

The clonal osteoblastic cell line MC3T3-E1 that is derived from newborn mouse calvaria has been used extensively as an *in vitro* model system for studying bone development. These cells undergo an orderly, time-dependent developmental sequence over three stages that are analogous to the respective stages of *in vivo* bone development. Nephronectin is an extracellular matrix protein known to be highly expressed in the long bone. This study was initiated by examination of the role of nephronectin in osteoblast differentiation and we discovered that ectopic expression of nephronectin promoted osteoblast differentiation. In the *in vitro* model system for bone development, ALP activity was used as a marker for osteoblast differentiation. Only the differentiated osteoblasts would stain pink/red for ALP expression. There was a significant difference in ALP expression between MC3T3-E1 cells transfected with the empty vector and those expressing full-length nephronectin. The function of endogenous nephronectin was confirmed by knocking down nephronectin expression with the siRNA approach.

We then investigated the mechanisms that could be involved in the regulation of nephronectin-mediated action. MiRNAs are known to bind to the 3′UTRs of genes to regulate their expression post-transcriptionally. Since nephronectin bears a long 3′UTR, we hypothesized that nephronectin expression is regulated by miRNAs. To test this hypothesis, we engineered a fragment of the nephronectin 3′UTR into the nephronectin expression construct. As expected, MC3T3-E1 cells stably transfected with nephronectin bearing the 3′UTR fragment (Npnt+3′) differentiated in lower levels as compared with those transfected with nephronectin alone in the early stages of differentiation. These results suggested that some endogenous miRNAs might have interacted with the 3′UTR fragment and repressed nephronectin expression resulting in a lower level of osteoblast differentiation. Nevertheless, Npnt+3′-transfected cells still differentiated at higher levels than the mock-transfected cells suggesting incomplete repression of ectopically transfected nephronectin. Unexpectedly, on examining the Npnt+3′-transfected cells over the three stages of bone development, we observed that the initial delay in osteoblast differentiation was followed by an equal rate of differentiation, that finally resulted in the Npnt+3′-transfected cells exhibiting significantly higher levels of osteoblast differentiation. This pattern was evident in bone nodule formation as well: after an initial delay in bone nodule formation, the Npnt+3′-transfected cells produced significantly higher densities of bone nodules than the nephronectin-transfected cells.

We hypothesized that over-expression of the 3′UTR might have attracted certain miRNAs binding to this fragment and, thereby making them unavailable for targeting some glycosylation-associated enzymes, which are essential for glycosylation of nephronectin. This in turn would increase the activity of glycosylation-associated enzymes, leading to produce more glycosylated nephronectin. Consequently, more nephronectin is secreted to promote osteoblast differentiation. To test this hypothesis, we investigated the differences in glycosylation of the nephronectin core protein in the presence and absence of the nephronectin 3′UTR using the two stable cell lines transfected with nephronectin or Npnt+3′. Western blot analysis showed that the Npnt+3′-transfected cells had a much higher level of glycosylated nephronectin compared with the nephronectin-transfected cells. These results therefore support our hypothesis.

The post-transcriptional regulation of nephronectin by miRNA was then examined. The use of bioinformatics algorithms showed that the 3′UTR of nephronectin was a potential target of miRNA *miR-378*. To examine the effect of *miR-378*, we generated a construct expressing dual fragments of pre-miR-378. Stable over-expression of the miR-378 construct strongly inhibited MC3T3-E1 differentiation. We have previously demonstrated that miR-378 enhances cell survival and tumor growth [53]. The report presented here that miR-378 inhibits cell differentiation is in consistent with our previous finding.

To examine the probability that translation of nephronectin was repressed by *miR-378*, we performed a number of co-transfection experiments including transient transfection and stable transfection of Npnt+3′ with the miR-378 construct in MC3T3-E1 osteoblasts and Hek293 cells. Unexpectedly, co-transfection of Npnt+3′ with miR-378 not only promoted nephronectin glycosylation, but also enhanced production of nephronectin, perhaps through enhanced translation, while miR-378 showed little effect on nephronectin (no 3′UTR) expression, as expected. We could explain this phenomenon by the fact that nephronectin might not be a true target of miR-378 although the sequence in its 3′UTR matches well with the sequence of miR-378. The ability to reliably identify the targets of a given miRNA has been known to be a major problem in the miRNA field. Most of the computational predictions of miRNA targets focus on base pairing between the miRNA “seed” region and its target mRNA. Despite the presence of the same target sites, it remains unclear why miR-378 was able to repress luciferase activity while the nephronectin 3′UTR when placed in the luciferase construct was not able to repress nephronectin expression. Zhao and co-workers have presented evidence for target selectivity which depends on the free energy surrounding the target site [60]. They proposed that a stable environment with lower free energy may not be accessible to the miRNA silencing complex as compared with the one with higher free energy (or unstable environment). We have also found that the secondary structure of the common miRNA binding site and its flanking regions is normally located in an unstable region with a multi-branching loop-like RNA structure [8]. How the average free energy flanking regions in a miRNA binding site affect targeting by a particular miRNA as well as the efficiencies of miRNA repression of gene expression await further investigation.

Bearing this in mind, we propose that binding of miR-378 to the 3′UTR of nephronectin not only did not exert direct repression of nephronectin synthesis, but it also created a secondary structure which is less accessible to other miRNAs which normally target nephronectin and regulate its expression. This space hindrance resulted in increased synthesis of nephronectin. Furthermore, the binding interaction of miR-378 with nephronectin 3′UTR would reduce free miR-378 for targeting nephronectin glycosylation-associated enzyme(s). As a consequence, the enzyme(s) expression would be enhanced resulting in increased nephronectin glycosylation and product secretion. We have previously demonstrated that glycosylation is an essential step for product secretion [57]–[59]. Concurrent with increased nephronectin protein synthesis, glycosylation, and secretion was the inhibition of proliferation and promotion of differentiation in MC3T3-E1 cells that were co-transfected with miR-378 and the Npnt+3′ construct.

To test whether some glycosylation-associated enzymes were affected, total proteins in MC3T3-E1 cells stably transfected with nephronectin or Npnt+3′ were subjected to proteomic and computational analyses. Indeed, GalNT7, an enzyme known to glycosylate proteins, was up-regulated in Npnt+3′-transfected cells and was downregulated by miR-378 expression. These results explained why expression of nephronectin 3′UTR was able to promote MC3T3-E1 differentiation and bone nodule formation in the late stages. However, it is not clear why these phenomena were not seen in the early stages. It is possible that the over-expressed nephronectin, although not secreted due to lack of glycosylation, interfered with some signalling molecules inside the cells, playing a role in cell differentiation. This awaits further investigation.

Taken together, we have reported a mechanism by which a miRNA can function in promoting protein expression in two ways. It can be achieved by binding to the 3′UTR and resulting in space hindrance preventing easy access of other miRNAs, thereby protecting the mRNA from being regulated post-transcriptionally. Secondly, a miRNA can promote protein expression by binding to the 3′UTR and freeing the true target of the miRNA, thereby increasing the levels of the target, which in turn enhances protein modification and secretion.

## Materials and Methods

### Materials

Dulbecco's modified Eagle's medium (DMEM), alpha modified Eagle's medium (AMEM), fetal bovine serum (FBS), trypsin/EDTA, Lipofectamine 2000, geneticin (G418), hygromycin were purchased from Wisent Inc. T4 DNA ligase, platinum *Pfx* DNA polymerase, SuperScript II reverse transcriptase, DH5α, and tissue culture supplies were purchased from Invitrogen. Restriction enzymes were purchased from New England Biolabs. RedTaq DNA polymerase, anti-actin and HRP-conjugated secondary antibodies, de-glycosylation enzymes (PNGase F, O-glycosidase, sialidase, and keratanase), horseradish peroxidase-conjugated goat anti-mouse IgG secondary antibody, and other general chemicals were purchased from Sigma. Tissue culture plates were purchased from Sarstedt (Montreal, QC, Canada). ECL Western blot detection kit was from Amersham Life Science (Baie d'Urfé, QC, Canada). The MC3T3-E1 cell line was obtained from the American Type Culture Collection (Rockville, MD).

### Cell culture

MC3T3-E1 and Hek293 cells were maintained in alpha-MEM and DMEM, respectively, supplemented with 10 (v/v) % fetal bovine serum, 100 units/ml penicillin, and 100 µg/ml streptomycin. Plasmid transfection was done using Lipofectamine 2000 according to the manufacturer's instruction. Transfected cells were selected under growth medium with 1 mg/ml G418 and were stable for more than one month before experiments.

Cell proliferation assays for MC3T3-E1 cells were performed for 2 weeks. In brief, 10^4^ cells were seeded on 12-well plates. The cells were harvested at indicated time points by trypsin/EDTA and then resuspended in 1 ml culture medium. After centrifugation, cell pellets were resuspended in 1% FBS containing 70% ethanol and kept at 4°C until analysis. Prior to use, the cell suspensions were exchanged into 1 ml fetal bovine serum (PBS) and the cell number was counted with a flow cytometer (FACSCalibur, BD bioscience, NJ, USA).

Differentiation of MC3T3-E1 osteoblast cells was carried out by incubation in differentiation medium (50 µg/ml ascorbic acid, 10 mM beta-glycerophosphate, and 10^−8^ M dexamethasone in alpha-MEM). The medium was changed every 2–3 days. Cell differentiation was identified by ALP staining. In general, differentiated cell layers were washed with PBS and fixed with neutral formalin (10% formaldehyde in PBS) for 15 min. After removal of fixation solution and successive incubation in distilled water for 15 min, the cell layers were stained with a staining solution (1 mg/ml Naphthol AS-MX-PO4; 6 mg/ml fast red violet LB salt, in 10 mM Tris-HCl, pH 8.0) at 37°C for 45 min. After removal of staining solution and rinsing 3 times with water, the stained cells were kept in distilled water for microscopic examination.

### Construct generation

The nephronectin cDNA was a kind gift of Dr. Louis. F. Reichardt. The primer sets used to amplify the two constructs of nephronectin are as follows. Npnt-N (5′cccgggctcgagtggcccaggcaaatagtttct) and Npnt-C (5′cccgggtctagatcagcagcgacctcttttcaa) were used to clone nephronectin coding sequence. Npnt-N and Npnt-end (5′cccggg**tctaga**aggaaattccatgcccaccct) were used to clone nephronectin coding sequence plus the 3′UTR. MAM-N (5′cccggg**ctcgag**gtgtatatccccaaagtc) and Npnt-C were used to cloned the fragment containing the MAM domain. A link protein leading peptide (LP) was attached to the N-terminus of the construct to allow product secretion and detection by the monoclonal antibody 4B6 [61], [62]. The sequences of the generated constructs were confirmed by restriction digestion and sequencing.

A luciferase reporter vector (pMir-Report; Ambion) was used to generate luciferase constructs containing either GalNT7 3′UTR or mutant GalNT7 3′UTR. The 3′UTR of GalNT7 was cloned using two primers, GalNT7-SacI (5′cccggg**gagctc**gcccttgttagattaggccttata) and GalNT7-MluI (5′gggccc**acgcgt**catttgcaatatttggttttcctaa), by PCR. The PCR products were then digested with SacI and MluI and the fragment was inserted into a SacI- and MluI-digested pMir-Report Luciferase plasmid (Ambion), to obtain a luciferase construct, luc-Gal-UTR. The mutant construct was generated with two primers, GalNT7-SacI and GalNT7-MluI-mut (5′gggccc**acgcgt**catttgcaatatttggttt**—––** aggaaatagg) using the same method.

To generate a small interfering RNA against nephronectin, two primers (5′ccaggtagatctaggactgaactgtgtgtatattcaagagtatacacacagttcagtccgactgccgaccagcagagcag and 5′gggctaaagcttaaaaaacagtaccactacacgagtagagaacattactcgtgtagtggtactgctgctctgctggtcggcagtc) were designed for PCR. The PCR product was digested with BglII and HindIII and inserted into the BglII- and HindIII-digested BluGFP vector [53], resulting in a construct expressing siRNA against nephronectin.

The construct for miR-378 was designed by us and synthesized by Top Gene Technologies (Montreal, Canada). In brief, the pre-miRNA of miR-378 was ligated into the mammalian expression vector BluGFP that contains a Bluescript backbone, a CMV promoter driving the GFP, and a promoter driving the expression of miR-378 as previous described [53], [63].

### Western blot and proteomic analysis

For western blot analysis, cells were harvested by 10 mM EDTA at 60–80 % confluence and washed three times with PBS using centrifugation. The cells (1×10^6^ cells) were disrupted and kept on ice for 15 min. The lysate was then centrifuged at maximum speed using a desktop centrifuge at 4 degrees for 10 min. Protein concentrations were quantified by a protein assay kit (Bio-Rad Laboratories Ltd., ON, Canada). Protein lysate (100 µg) was subjected to SDS-polyacrylamide gel electrophoresis (PAGE) and the separated proteins were transferred onto a nitrocellulose membrane followed by blocking and incubation in a primary monoclonal antibody overnight at 4 degrees. After washing, the membrane was incubated with HRP-conjugated goat-anti-mouse secondary antibody for 1 hour at room temperature followed by ECL detection.

For proteomic analysis, the cells were cultured for three days under normal growth conditions. The cells were detached from the culture dishes by 1 mM EDTA and then washed three times with PBS. Cell pellets were then delivered on dry ice for proteomic analysis by WEMB Biochem Inc (Richmond Hill, ON, Canada).

### Characterization of protein glycosylation

To characterize the type of post-translational modification of nephronectin, the conditioned medium containing fully glycosylated nephronectin was digested by different de-glycosylation enzymes. The size of the resulting protein was then observed by western blot. The conditioned medium (30 ul) was treated with 10 µl enzyme solution containing 2 units PNGase F (to release *N*-glycan), 2.5 units *O*-glycosidase (to release *O*-glycan), 0.01 units sialidase (to release terminal sialic acid) or 0.1 units keratinase (to release keratin sulfate) at 37°C overnight. To completely remove the *O*-glycan from the protein core, the combined digestion by *O*-glycosidase and sialidase was achieved by the same incubation conditions.

### Statistical Analysis

The results (mean values±SD) of all experiments were subjected to statistical analysis by the *t*-test. The level of significance was set at *P*<0.05.

## Supporting Information

Figure S1 Effects of nephronectin on MC3T3-E1 cell activities. (A) Time course analysis showing the different effects of Npnt and Npnt+3′ transfections on MC3T3-E1 differentiation: early stages, Npnt>Npnt+3′; late stages, Npnt<Npnt+3′. (B) The cell lines were cultured under nodule-inducing conditions over the three different phases of osteoblast development. Bone nodules formed on day 56 are shown after silver nitrate staining for the detection of bone nodules. The existence of miR-378 slightly enhanced cell proliferation in Npnt-transfected cells (C), but inhibited cell proliferation in Npnt+3′-transfected cells (D).
(1.52 MB PPT)

Figure S2 Targeting of GalNT7 by miR-378. (A) A fragment of GalNT7 3′UTR was inserted into the luciferase report vector pMir-Report producing a construct named luc-Gal-UTR. The potential miR-378 target sequence was labelled in blue. Mutations labelled in red color were generated in the miR-378 target sequence producing a mutant construct named luc-Gal-mut. (B) MT-1 cells stably transfected with GFP and miR-378 were immunostained with anti-GalNT7 antibody, followed by confocal microscopic analysis. While both types of cells expressed GFP (green), miR-378-transfected cells expressed lower levels of GalNT7 than the GFP-transfected cells (red). As a result, the merged color of miR-378-transfected cells were less yellow. (C) MC3T3 cells were transiently transfected with siRNAs or a random sequence serving as a negative control. Silencing of GalNT7 expression were analyzed by real-time PCR.
(2.35 MB PPT)

Table S1 (3.79 MB PPT)
