# Supplementary material for: MicroRNA miR-378 Regulates Nephronectin Expression Modulating Osteoblast Differentiation by Targeting GalNT-7
Source: PLoS One. 2009 Oct 21;4(10):e7535. doi: 10.1371/journal.pone.0007535 (PMC2760121; doi:10.1371/journal.pone.0007535)
Supplement: Figure S1 — Effects of nephronectin on MC3T3-E1 cell activities. (A) Time course analysis showing the different effects of Npnt and Npnt+3′ transfections on MC3T3-E1 differentiation: early stages, Npnt>Npnt+3′; late stages, Npnt<Npnt+3′. (B) The cell lines were cultured under nodule-inducing conditions over the three different phases of osteoblast development. Bone nodules formed on day 56 are shown after silver nitrate staining for the detection of bone nodules. The existence of miR-378 slightly enhanced cell proliferation in Npnt-transfected cells (C), but inhibited cell proliferation in Npnt+3′-transfected cells (D). (1.52 MB PPT) [file pone.0007535.s001.ppt]

## Slide 1
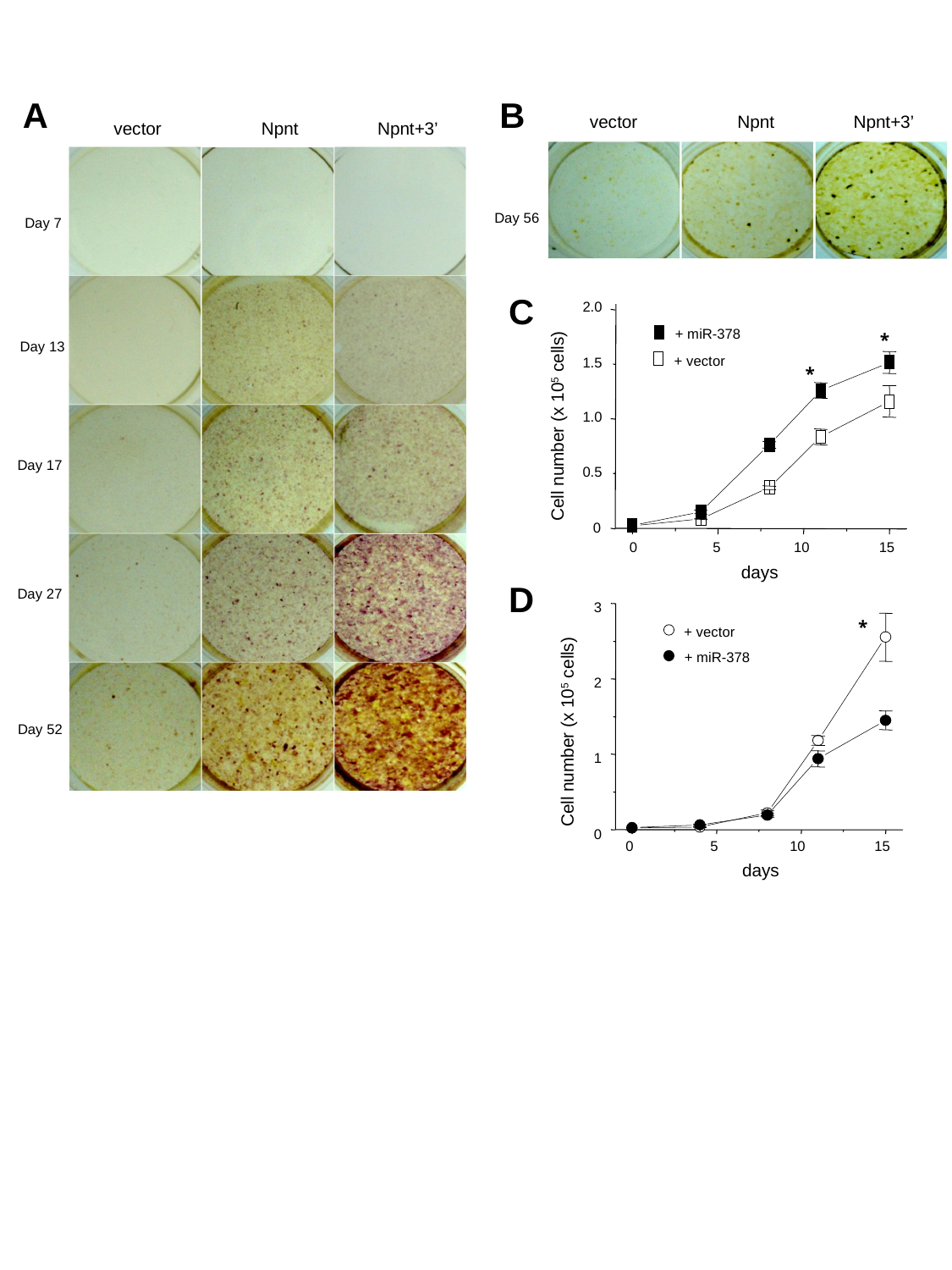

B
A
vector
Npnt
Npnt+3’
vector
Npnt
Npnt+3’
Day 56
Day 7
C
2.0
*
+ miR-378
Day 13
+ vector
1.5
*
1.0
Cell number (x 105 cells)
Day 17
0.5
0
0
5
10
15
days
D
Day 27
3
*
+ vector
+ miR-378
2
Day 52
Cell number (x 105 cells)
1
0
0
5
10
15
days
