# Supplementary material for: MicroRNA miR-378 Regulates Nephronectin Expression Modulating Osteoblast Differentiation by Targeting GalNT-7
Source: PLoS One. 2009 Oct 21;4(10):e7535. doi: 10.1371/journal.pone.0007535 (PMC2760121; doi:10.1371/journal.pone.0007535)
Supplement: Figure S2 — Targeting of GalNT7 by miR-378. (A) A fragment of GalNT7 3′UTR was inserted into the luciferase report vector pMir-Report producing a construct named luc-Gal-UTR. The potential miR-378 target sequence was labelled in blue. Mutations labelled in red color were generated in the miR-378 target sequence producing a mutant construct named luc-Gal-mut. (B) MT-1 cells stably transfected with GFP and miR-378 were immunostained with anti-GalNT7 antibody, followed by confocal microscopic analysis. While both types of cells expressed GFP (green), miR-378-transfected cells expressed lower levels of GalNT7 than the GFP-transfected cells (red). As a result, the merged color of miR-378-transfected cells were less yellow. (C) MC3T3 cells were transiently transfected with siRNAs or a random sequence serving as a negative control. Silencing of GalNT7 expression were analyzed by real-time PCR. (2.35 MB PPT) [file pone.0007535.s002.ppt]

## Slide 1
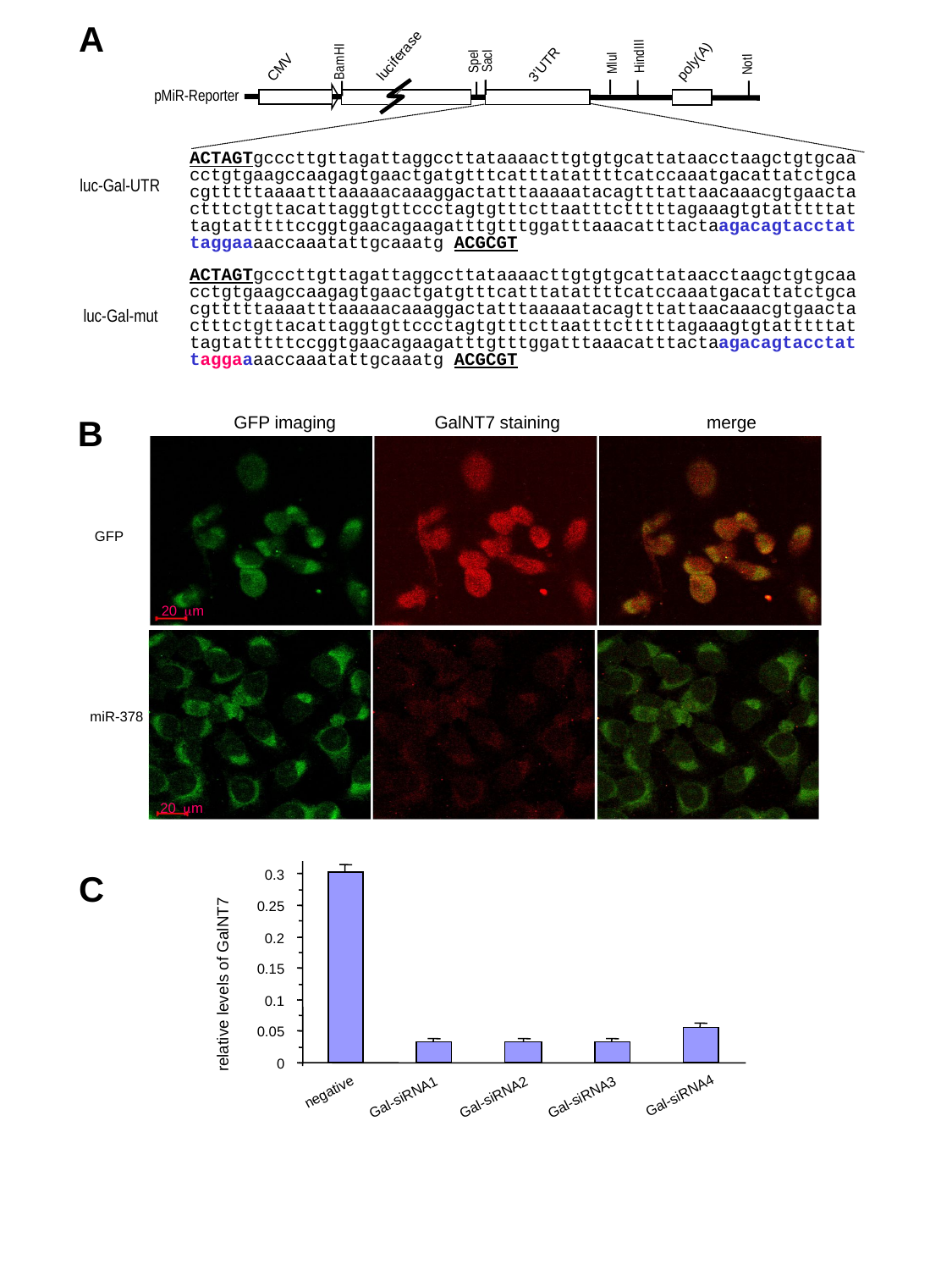

A
luciferase
HindIII
3’UTR
SacI
SpeI
BamHI
 poly(A)
MluI
NotI
CMV
pMiR-Reporter
ACTAGTgcccttgttagattaggccttataaaacttgtgtgcattataacctaagctgtgcaacctgtgaagccaagagtgaactgatgtttcatttatattttcatccaaatgacattatctgcacgtttttaaaatttaaaaacaaaggactatttaaaaatacagtttattaacaaacgtgaactactttctgttacattaggtgttccctagtgtttcttaatttctttttagaaagtgtatttttattagtatttttccggtgaacagaagatttgtttggatttaaacatttactaagacagtacctattaggaaaaccaaatattgcaaatg ACGCGT
luc-Gal-UTR
ACTAGTgcccttgttagattaggccttataaaacttgtgtgcattataacctaagctgtgcaacctgtgaagccaagagtgaactgatgtttcatttatattttcatccaaatgacattatctgcacgtttttaaaatttaaaaacaaaggactatttaaaaatacagtttattaacaaacgtgaactactttctgttacattaggtgttccctagtgtttcttaatttctttttagaaagtgtatttttattagtatttttccggtgaacagaagatttgtttggatttaaacatttactaagacagtacctattaggaaaaccaaatattgcaaatg ACGCGT
luc-Gal-mut
B
 GFP imaging
 GalNT7 staining
 merge
 GFP
 20 μm
 miR-378
 20 μm
C
0.3
0.25
0.2
0.15
relative levels of GalNT7
0.1
0.05
0
negative
Gal-siRNA4
Gal-siRNA1
Gal-siRNA2
Gal-siRNA3
