# Supplementary figures and images for: MicroRNA miR-378 Regulates Nephronectin Expression Modulating Osteoblast Differentiation by Targeting GalNT-7
Source: PLoS One. 2009 Oct 21;4(10):e7535. doi: 10.1371/journal.pone.0007535 (PMC2760121; doi:10.1371/journal.pone.0007535)

## Slide 1
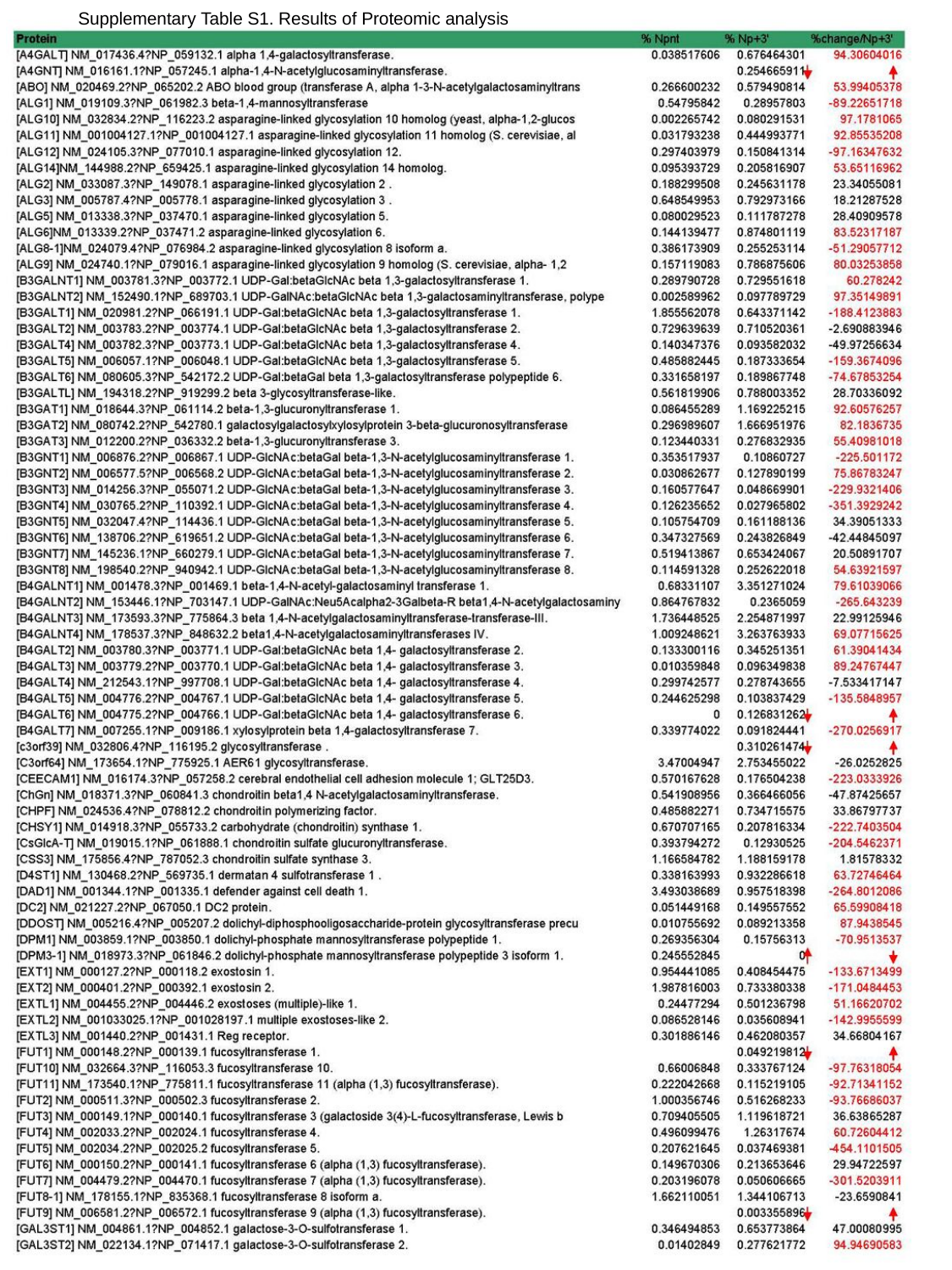

Supplementary Table S1. Results of Proteomic analysis

## Slide 2
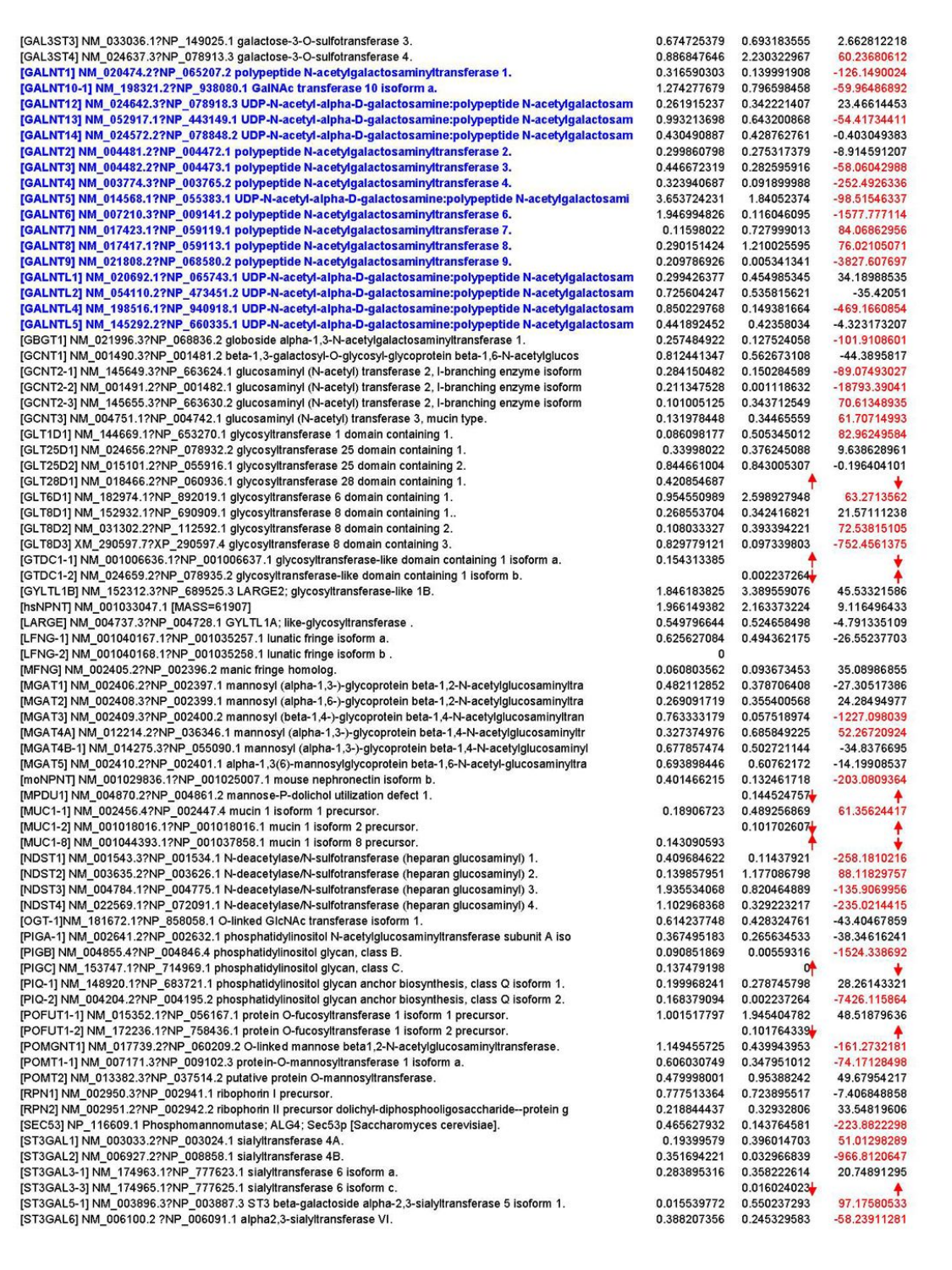

## Slide 3
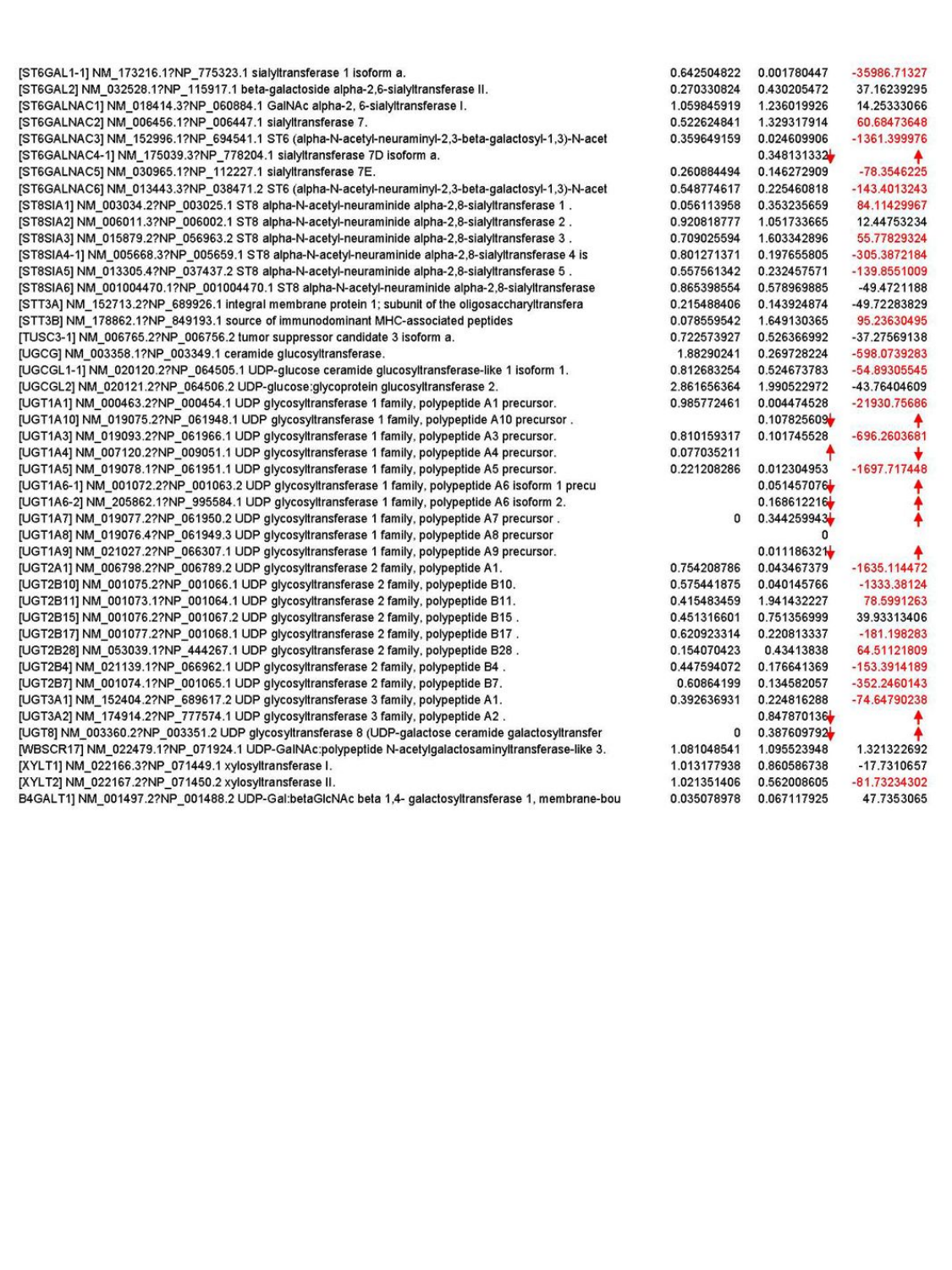

Supplement: Table S1 — (3.79 MB PPT) [file pone.0007535.s003.ppt]
